# Therapeutic Potential of Mesenchymal Stem Cell-Derived Exosomes as Nanomedicine for Peripheral Nerve Injury

**DOI:** 10.3390/ijms25147882

**Published:** 2024-07-18

**Authors:** Qicheng Li, Fengshi Zhang, Xiaoyang Fu, Na Han

**Affiliations:** 1 Department of Trauma and Orthopedics, Peking University People’s Hospital, Beijing 100044, China; qicheng.li@pku.edu.cn (Q.L.); xmx066@pku.edu.cn (F.Z.); wyw761398420@163.com (X.F.); 2 Key Laboratory of Trauma and Neural Regeneration, Peking University, Beijing 100044, China; 3 National Center for Trauma Medicine, Beijing 100044, China

**Keywords:** peripheral nerve injury, nerve regeneration, mesenchymal stem cells, exosomes, biomaterials

## Abstract

Peripheral nerve injury (PNI) is a complex and protracted process, and existing therapeutic approaches struggle to achieve effective nerve regeneration. Recent studies have shown that mesenchymal stem cells (MSCs) may be a pivotal choice for treating peripheral nerve injury. MSCs possess robust paracrine capabilities, and exosomes, as the primary secretome of MSCs, are considered crucial regulatory mediators involved in peripheral nerve regeneration. Exosomes, as nanocarriers, can transport various endogenous or exogenous bioactive substances to recipient cells, thereby promoting vascular and axonal regeneration while suppressing inflammation and pain. In this review, we summarize the mechanistic roles of exosomes derived from MSCs in peripheral nerve regeneration, discuss the engineering strategies for MSC-derived exosomes to improve therapeutic potential, and explore the combined effects of MSC-derived exosomes with biomaterials (nerve conduits, hydrogels) in peripheral nerve regeneration.

## 1. Introduction

The challenge of addressing limb paralysis and functional impairments resulting from peripheral nerve injury is a global clinical concern [1]. In the United States, the average care cost for a patient with upper limb peripheral nerve injury reaches USD 45,000, exhibiting an annual growth rate of 9.59%. Consequently, the annual healthcare expenditure for treating diverse conditions arising from peripheral nerve injury amounts to USD 150 billion [2]. The current therapeutic approaches for peripheral nerve injury depend on the extent of nerve damage, with autologous nerve transplantation representing the gold standard for the restoration of lost neural tissue associated with peripheral nerve injury [3]. However, limitations in the source of donor nerves and the potential loss of donor site nerve function restrict the application of nerve transplantation [4]. Currently, researchers focus on developing novel methods to promote functional and phenotypic changes in Schwann cells (SCs) at the nerve injury site, restore the neurovascular barrier, facilitate rapid clearance of myelin debris, and reduce inflammatory factor levels.

Mesenchymal stem cells (MSCs) have garnered considerable attention in the field of nerve injury repair [5,6]. Previous reports have indicated that transplantation of MSCs, in conjunction with nerve conduit, into the site of sciatic nerve lesion stimulated axonal growth, promoted myelin sheath formation, and facilitated recovery from denervation-induced muscle atrophy [7,8]. Although MSCs can differentiate into Schwann cell-like cells in vitro under specific culture conditions for neural tissue repair, cell transplantation does not lead to Schwann cell differentiation within the injury microenvironment in vivo [9,10]. These results suggested that the therapeutic efficacy of transplanted MSCs depends on the indirect regeneration of endogenous SCs via paracrine mechanisms rather than transdifferentiation. Furthermore, studies have documented a decrease in cell viability and uncontrolled differentiation of MSCs during transplantation [11,12], which prompted researchers to explore innovative strategies for cell-free therapies in the context of peripheral nerve injury.

Exosomes, ranging in diameter from 30 to 150 nm, represent a subset of cell-derived vesicles actively secreted into surrounding bodily fluids via the paracrine pathway [13,14]. Recent investigations have underscored the significance of exosomes in the diagnosis and treatment of diverse diseases, such as neurodegenerative disorders [15], nervous system tumors [16], and nerve injuries [17]. These nanovesicles carry a variety of cargo from parent cells, encompassing proteins, nucleic acids, and lipids [18]. Engineering approaches have been explored to enhance exosomes yield and optimize therapeutic potential, thereby ensuring a sustained, precise, and effective impact on recipient cells. Accumulating evidence indicates that exosomes derived from mesenchymal stem cells (MSCs) exhibit remarkable functions in regenerative medicine, such as regulating inflammation [19], facilitating cell proliferation [20], enhancing vascular regeneration [21], and inhibiting cell apoptosis [22]. Therefore, this review delineates the reparative advancement of MSC-derived exosomes in peripheral nerve injury. Additionally, we explore engineering strategies to enhance the therapeutic potential of MSC-derived exosomes and their combined application with nerve conduits, shedding light on potential synergies in neural tissue repair.

## 2. Overview of Peripheral Nerve Injury

Peripheral nerve injury is a complex process involving multiple factors, and ongoing efforts are directed toward continually clarifying its pathophysiological mechanisms. Peripheral nerve injuries are divided into three categories based on severity: neurapraxia, axonotmesis, and neurotmesis [23]. Neurotmesis represents the most severe type of peripheral nerve injury, involving the complete transection of the peripheral nerve connection, presenting substantial challenges to nerve regeneration and functional recovery [24]. Following peripheral nerve injury, pathological alterations manifest in the injured site, nerve stumps, neurons, and target organs [25]. Neuronal bodies exhibit swelling, nuclear deviation, and chromatolysis, while distal segments of nerve fibers undergo Wallerian degeneration (WD) [26,27]. Nerve terminal organs, such as motor endplates and sensory corpuscles, undergo degeneration and denervation [28]. Concomitantly, mature Schwann cells dedifferentiated into repair Schwann cells, participating in the recruitment of macrophages, engulfment of myelin debris, and the formation of *Büngner* bands [29,30].

While mild peripheral nerve injury can be alleviated through self-repair mechanisms, severe peripheral nerve discontinuity injury and mixed nerve injury require external intervention. Clinically, autologous nerve transplantation is the preferred strategy for the reconstruction of long peripheral nerve defects, but it is constrained by size mismatch, low graft availability, and risk of neuroma formation [31]. In recent years, significant progress has been made in repairing peripheral nerve injury through tissue engineering techniques. Mesenchymal stem cells and their secretions have emerged as promising candidates for peripheral nerve regeneration due to their outstanding functional contributions to tissue regeneration [5,32].

## 3. Mesenchymal Stem Cells

**Figure 1:**
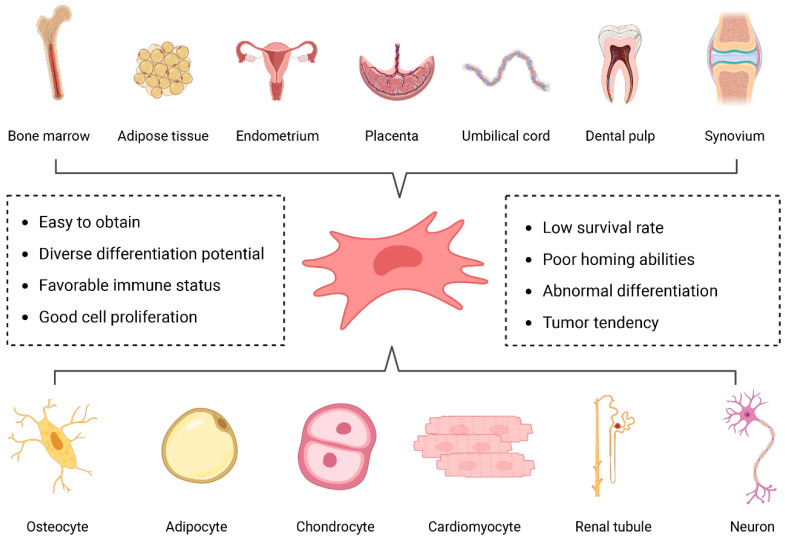
Source and differentiation potential of mesenchymal stem cells. MSCs are derived from a variety of tissues, including bone marrow, adipose tissue, endometrium, placenta, umbilical cord, dental pulp, and synovium, which can differentiate into osteocytes, adipocyte, chondrocytes, cardiomyocytes, renal tubules, neurons, and Schwann cells through special induction. Figures created with Biorender.com.

Mesenchymal stem cells (MSCs) are a subtype of adult stem cells derived from the mesodermal layer during early embryonic development [33], exhibiting self-renewal capacity, multipotent differentiation potential, and the ability to maintain their biological characteristics after in vitro scale-up expansion [34]. MSCs are found in various tissues, such as bone marrow, adipose tissue, dental pulp, placenta, and umbilical cord [35] (Figure 1). Under the influence of different induction culture media in vitro, MSCs can differentiate into cell-specific lineages, including chondrocytes, osteoblasts, adipocytes, neurons, and glial cells [36] (Figure 1). For instance, when cultured with media pre-incubated with cerebrospinal fluid to provide appropriate neurotrophic factors, MSCs can differentiate into neurons [37]. Furthermore, Wang et al. [38] reported that bone mesenchymal stem cells (BMSCs) can differentiate into the Schwann cell phenotype in vitro after being cocultured with peripheral nerve extracts from the distal segment of degenerated rat sciatic nerve. However, the application of MSCs is accompanied by certain disadvantages, such as low survival rate of transplantation, poor migration and homing abilities, abnormal differentiation, and tumor tendency [39].

## 4. Biogenesis of Exosomes

**Figure 2:**
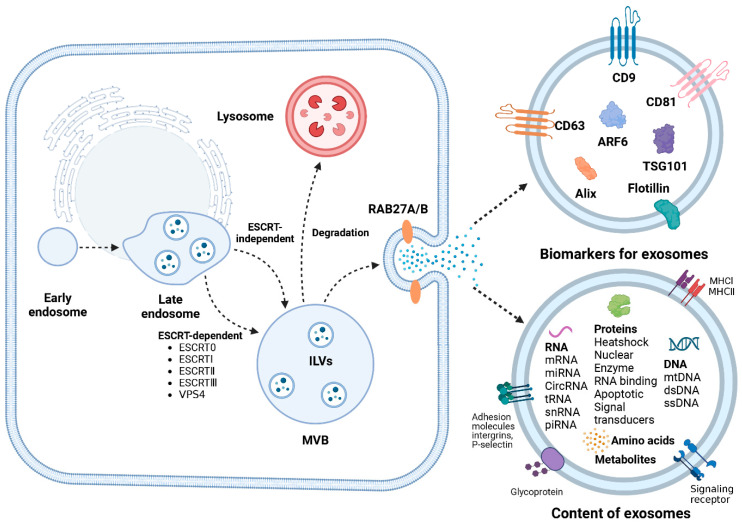
Biogenesis and hallmarks of exosomes. (1) Biogenesis of living cell-derived exosomes originates from the endocytosis pathway. (2) Exosomes contain specific proteins, such as CD9, CD63, CD81, and TSG101. (3) Exosomes carry a variety of contents, including RNA, proteins, DNA, amino acids, and metabolites. Figures created with Biorender.com.

In 1987, Johnstone et al. [40] first described extracellular “exosomes” as a new extracellular vesicle (EV) subtype. Since then, the origin and functions of exosomes have garnered widespread attention. Studies have revealed that exosomes’ biogenesis begins in the endosomal pathway, where continuous invagination of the limiting membrane during early endosome maturation results in the formation of intraluminal vesicles (ILVs) [41] (Figure 2). During this process, proteins, nucleic acids, and lipids are selectively sorted into ILVs. Subsequently, ILVs further mature into multivesicular bodies (MVBs), with the endosomal sorting complex required for transport (ESCRT) playing a pivotal role in this highly intricate process [42]. The ESCRT machinery comprises five complexes, ESCRT-0, ESCRT-I, ESCRT-II, ESCRT-III, and vacuolar protein sorting 4 (VPS4), along with the Bro1 protein family (ALIX, HDPTPT) [43]. ESCRT facilitates the budding of ILVs and their release into the lumen of MVBs. ESCRT-0, consisting of HRS and STAM proteins, each containing two ubiquitin-binding domains (UBD), facilitates the capture of ubiquitinated cargo proteins for aggregation [44]. Additionally, ESCRT-I recruits ESCRT-II to form a stable vesicle neck and regulates the release of vesicles; ESCRT-II mediates ESCRT-III assembly and regulates cytoskeleton separation [45]. ESCRT-III lacks similar structural motifs; instead, it assembles into a filamentous structure associated with the membrane and catalyzes membrane scission and factor recycling through the VPS4 complex, ultimately forming MVBs [46]. The biogenesis of exosomes is not exclusively mediated by the ESCRT pathway, as complete disruption of ESCRT function does not eliminate ILV formation. Non-ESCRT-dependent mechanisms for MVB formation, particularly the neutral sphingomyelinase (nSMase) pathway, have been extensively reported [47].

The ultimately formed multivesicular body (MVB) undergoes regulation by ISGylation, with a fraction directed towards lysosomal degradation and another fraction transported to the plasma membrane mediated by Rab GTPase [48,49]. Subsequently, late endosomes fuse with specific regions of the plasma membrane, facilitating the release of exosomes from the MVB into the extracellular microenvironment through either SNARE-dependent or independent pathways.

## 5. Isolation of Exosomes

Generally, exosomes are widely present in a variety of body fluids, such as amniotic fluid, blood, breast milk, cerebrospinal fluid, saliva, and urine [50], which can be extracted and purified based on their different characteristics and research objectives for applying in diagnosis and treatment [51,52] (Table 1). Ultracentrifugation is a widely used protocol for exosome isolation, which is considered the gold standard method. In one study, three commercial kits were used to isolate culture supernatants compared to ultracentrifugation as a control [53]. Although ultracentrifugation possessed potential advantages of easy operation and high purity of products, the disadvantages of low yield, time consumption and protein aggregation still limited its large-scale application. Moreover, precipitation-based methods were used to tie up water molecules and force less-soluble components out of samples. Polyethylene glycol is a kind of hydrophilic polymer, which can surround exosomes to allow exosomes’ precipitation at low-speed centrifugation. This method is simple and increases exosome yield, but the purity of the exosomes is hampered by other proteins precipitated together with exosomes [54]. To improve the efficiency of exosome isolation, some studies have sought alternative innovative separation techniques. For instance, Shi et al. [55] developed an insulator-based dielectrophoresis (DEP) method for the non-specific and rapid capture of exosomes. Based on the dielectric properties of vesicles, exosomes were captured and pre-concentrated from healthy human plasma in 2 min using a DEP device with a charged nanopipette. The results showed that the size of the nanopipette pore did not affect separation efficiency, but different geometry of the same pore size produced inconsistent results. This indicated that there are still potential factors affecting the extraction of exosomes using charge-based methods. Additionally, microfluidic-based techniques are novel isolation approaches, which utilize the physical and biochemical characteristics of exosomes to offer refined microcarriers for exosome preparation. This technology integrates advanced techniques, such as acoustics, electrophoresis and electromagnetic operations with conventional methods (size, density and immunoaffinity). It increases the production of high-purity exosomes and has greater efficiency of exosome isolation, while being unavailable for large-scale production of exosomes [56]. Currently, these strategies for exosome isolation are restricted to laboratory research because of their potential shortcomings. Therefore, the development of more efficient and scaled-up separation techniques for exosomes are driven forward for the clinical treatment of peripheral nerve injury.

## 6. Effects of MSC-Derived Exosomes on Regeneration of PNI

**Figure 3:**
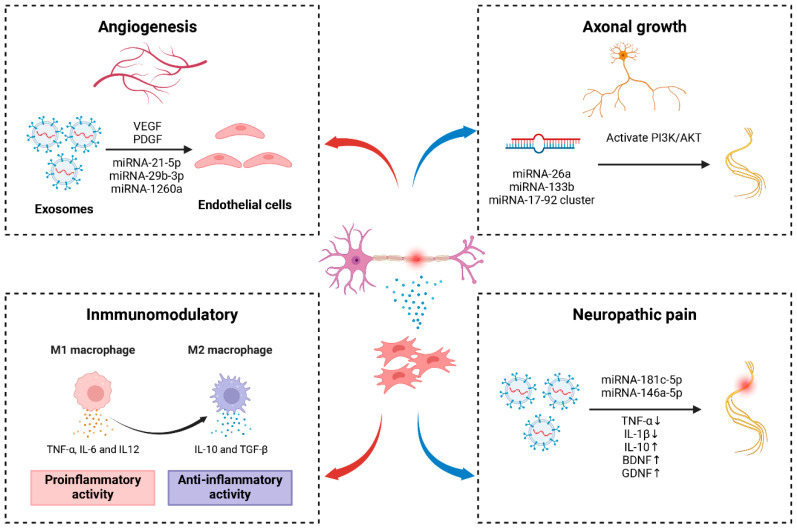
Exosomes derived from MSCs act on peripheral nerve injury, promoting angiogenesis, enhancing axonal growth, modulating neuroinflammation, and alleviating neuropathic pain. Figures created with Biorender.com.

Exosomes serve as the principal paracrine effectors of mesenchymal stem cells, facilitating intercellular communication, and maintaining the homeostatic and dynamic microenvironment [57,58]. When introduced into the site of peripheral nerve injury, exosomes derived from MSCs employ cellular internalization, receptor-ligand recognition, and membrane fusion mechanisms to enter recipient cells [59]. The process promotes angiogenesis and axonal regeneration at the site of peripheral nerve injury, modulates neuroinflammation, and alleviates neuropathic pain (Figure 3). The realization of these functions involves a diverse array of molecular mechanisms and signaling pathways (Table 2).

### 6.1. MSC Exosomes Promote Angiogenesis

After peripheral nerve injury, the disruption of the blood–nerve barrier is a crucial factor resulting in secondary damage to peripheral nerves [60,61]. The reconstruction of the vascular network provides a regenerative microenvironment for axonal growth, making the maintenance of vascular integrity a key therapeutic target for peripheral nerve regeneration [62,63]. Endothelial cells constitute a critical component of the vascular wall, with the ability to secrete various biologically active substances favorable to axonal growth. MSC-derived exosomes were rich in proteins associated with angiogenesis, such as vascular endothelial growth factor (VEGF) and platelet-derived growth factor-D (PDGF-D), which acted on endothelial cells, exerting a pro-angiogenic effect [64,65]. Moreover, MSC-derived exosomes transferred angiogenic miRNAs to human umbilical vein endothelial cells (HUVECs) to modulate the expression of miRNA target genes. For example, BMSC-derived exosomes can carry miRNA-1260a, miRNA-21-5p, and miRNA-29b-3p to enhance vascular regeneration [66,67,68]. These exosomal miRNAs entered endothelial cells and regulated multiple signaling pathways, such as AKT/eNOS and PI3K/Akt [21,66]. In peripheral nerve injury, exosomes derived from adipose-derived mesenchymal stem cells (ADMSC) towards the Schwann cell phenotype (dExo) can enhance antioxidative, angiogenic, anti-inflammatory, and axon growth properties, which are related to upregulated miRNAs in dExo, such as miRNA-132-3p and miRNA-199b-5p [69]. All these studies collectively suggest that exosome-mediated angiogenesis is beneficial for the repair of peripheral nerve injury.

### 6.2. MSC Exosomes Mediate Axonal Regeneration

Exosomes derived from neural or non-neuronal cells have been demonstrated to facilitate neurite outgrowth or enhance axonal regeneration in damaged neurons [70,71]. Currently, the mechanisms by which exosomes promote axonal regeneration remain unclear but are possibly attributable to the cargo carried by these vesicles, including miRNA, peptides, and various signaling molecules [72]. Exosomes from different sources of MSCs, such as BMSC and ADMSC, have been shown to promote the growth of dorsal root ganglion neuron axons [17,73]. Xie et al. [74] reported that exosomes derived from ADMSCs stimulate proliferation and migration of PC12 cells (rat pheochromocytoma cell line, model for the study of neuronal differentiation and neuroregeneration) by activating the PI3K/AKT pathway. In a recent study, exosomes from gingival mesenchymal stem cells (GMSCs) were found to enhance Schwann cell dedifferentiation, thereby promoting damaged axonal regeneration and functional recovery through activating gene expression of c-JUN [75].

Furthermore, MSC-derived exosomes carrying specific miRNAs promoted axonal regeneration compared to naturally secreted exosomes from MSCs. Previous investigations have demonstrated that exosomes derived from BMSCs can facilitate the survival and axonal regeneration of retinal ganglion cells following optic nerve crush injury in rats [76]. This pivotal effect relied on miRNA, as evidenced by the diminished therapeutic effects upon downregulation of the miRNA effector molecule Argonaute-2. Multifaceted studies have confirmed that exosomes derived from genetically engineered mesenchymal stem cells enriched with miR-26a, miR-133b, and miR-17-92 cluster increased axonal growth [77,78,79]. Therefore, the presence of bioactive molecules in MSC-derived exosomes associated with neural repair offers the potential for regulating the recovery of denervation atrophy, neuronal survival, and axonal growth.

### 6.3. MSC Exosomes Modulate Neuroinflammation

Neuroinflammation is one of the key factors in peripheral nerve injury and regeneration. When peripheral nerve injury occurs, the distal nerve myelinated SCs are dedifferentiated into repaired SCs, releasing a variety of chemokines and proinflammatory cytokines to recruit peripheral immune cells (such as circulating macrophages) to accumulate in the injured site [80]. Macrophages removed myelin fragments and avoided the inhibition of axon growth by residual axon fragments in the later stage of Wallerian degeneration. However, excessive inflammation hinders peripheral nerve regeneration. MSC-derived exosomes can communicate with the inflammatory microenvironment to exert neuroprotective effects and regulate complementary systems to promote nerve function recovery. Ti et al. [81] demonstrated that lipopolysaccharide (LPS) pre-treated MSCs can enhance M2 macrophage activation, upregulate anti-inflammatory factor expression, reduce inflammation infiltration, and effectively alleviate chronic inflammation. Further analysis revealed that LPS pre-treated exosomes exert their function by carrying miRNA, specifically let-7b, which participated in regulating the TLR4/NF-κB/STAT3/AKT signaling pathway. Moreover, Sun et al. [82] found the intravenous injection of exosomes secreted by human umbilical cord MSCs (hUCMSCs) can convert M1 macrophages into M2 macrophages. The conversion led to a downregulation of inflammatory cytokines, thereby alleviating inflammation in the damaged area, promoting tissue healing, and expediting the recovery of functional impairments. Collectively, these findings suggest exosome-mediated neuroinflammatory modulation for the repair of peripheral nerve injury.

### 6.4. MSC Exosomes Alleviate Neuropathic Pain

Neuropathic pain, arising from lesions or diseases affecting the central or peripheral nervous system, constitutes a refractory chronic condition [83]. Current therapeutic strategies for neuropathic pain, encompassing physical, pharmacological, and interventional approaches, exhibit limited efficacy [84]. MSC-derived exosomes present a promising pathway for alleviating chronic pain by mitigating inflammatory responses. Fan et al. [85] administered peripheral intravenous injections of BMSC-derived exosomes into diabetic model mice, significantly reducing mechanical and thermal pain thresholds. Bioinformatics analysis revealed an abundance of miRNAs targeting the TLR4/NF-κB signaling pathway within BMSC-derived exosomes, suggesting their potential role in suppressing inflammation and reducing neuropathic pain. Shiue et al. [86] demonstrated that intrathecal injection of exosomes extracted from MSCs significantly alleviated spinal nerve ligation (SNL)-induced neuropathic pain. In SNL rats, ipsilateral L5 spinal dorsal horn ganglia exhibited a marked increase in IL-10, brain-derived, and glial cell-derived neurotrophic factors compared to the control group. The expression of TNF-α and IL-1β decreased due to exosome-mediated inhibition of microglial and astrocytic cell activation. Similarly, Hsu et al. [87] observed significant relief of neuropathic pain induced by the SNL rat model following the injection of UCMSC-derived exosomes at the injury site. This intervention led to a reduction in TNF-α and IL-1β levels in the L5-6 dorsal root ganglia on the injured side, accompanied by an elevation in IL-10 and brain-derived neurotrophic factor levels, underscoring the role of exosomes in alleviating pain through the inhibition of inflammatory progression. In other studies, MSC-derived exosomal miRNAs, such as miR-181c-5p and miR-146a-5p, have also demonstrated potential in alleviating neuropathic pain [84,88].

**Table 2:** Mechanisms of MSC exosomes promoting peripheral nerve regeneration.

Source Cell	Exosomal Cargo	Signaling Pathway	Effects	References
ADMSCs	miRN-132-3p, miRNA-199b-5p	N/A	Protected SCs from oxidative stress, enhanced angiogenesis, promoted axonal growth	[69]
ADMSCs	N/A	PI3K/AKT signaling pathway	Promoted PC12 cell proliferation and migration, inhibited apoptosis	[74]
GMSCs	N/A	Expression of c-JUN key transcription factor	Promoted axonal regeneration and functional recovery, activated repair phenotype of SCs	[75]
MenSCs	N/A	PI3K/AKT signaling pathway	Against neuron injury induced by glutamate	[89]
BMSCs	miR-17-92 cluster	PI3K/protein kinase B/mechanistic target of rapamycin/glycogen synthase kinase 3β	Increased neural plasticity and functional recovery	[79]
BMSCs	Let-7a, miR-23a and miR-125b	TLR/NF-κB signaling pathway	Increased angiogenesis and nerve regeneration, suppressed proinflammatory cytokines	[85]

## 7. Strategies to Improve Therapeutic Function of MSC Exosomes

### 7.1. Pretreatment Approaches of Parental Cells

**Figure 4:**
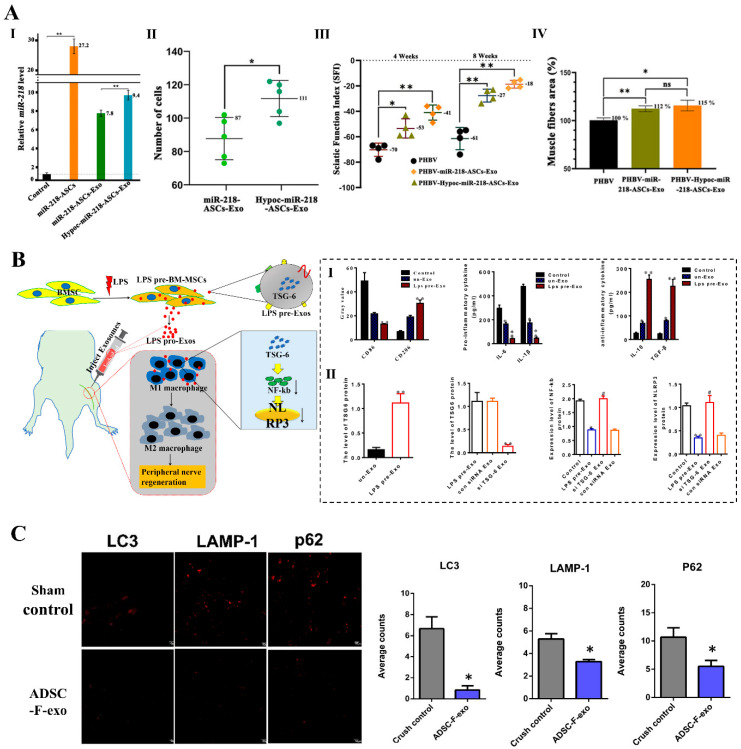
Exosomes produced from preconditioned MSCs enhanced peripheral nerve regeneration. (**A**) Exosomes isolated from the hypocapnia environment cultured ADMSCs upregulated miR−218 expression (**I**), promoted PC12 migration (**II**), and restored the regeneration of motor and nerve fibers (**III**,**IV**). Adapted with permission from [91], copyright 2022, Frontiers. (**B**) Exosomes derived from LPS-preconditioned MSCs accelerated the M2 macrophage polarization (**I**) and delivered TSG6 protein into macrophages to inhibit the expression of NF−κB and NLRP3 (**II**). Adapted with permission from [94], copyright 2022, Elsevier. (**C**) Exosomes secreted by ADMSC following FK506 stimulation reduced the expression of autophagy−related markers (LC3, P62, or LAMP-1) in the spinal segment after nerve crush injury. Adapted with permission from [97], copyright 2021, MDPI.

The quantity and function of natural exosomes produced by different MSCs are limited. Interestingly, source cells can regulate the production and bioactivity of exosomes with the change in microenvironment. The previous studies have shown that low gas (low oxygen, low carbon dioxide) in culture environment significantly influenced cellular physiological functions [90,91]. For example, ADMSCs cultured in a low carbon dioxide environment exhibited remarkable upregulation of miR-218 in the engineered exosomes, thereby promoting the activity of PC12 cells [91]. In vivo, hypocapnia-stimulated exosomes delivering miR-218 combined with bioscaffold facilitated the regeneration of motor and nerve fibers (Figure 4A). Additionally, cells cultured in a 3D system demonstrated a closer resemblance to in vivo conditions compared to traditional 2D culture methods, enhancing the paracrine effects of MSCs and optimizing exosome function [92]. For instance, hBMSCs cultured in 3D Synthemax II microcarriers in the PBS mini 0.1 L Vertical-Wheel bioreactor system resulted in a 2.5-fold increase in exosome secretion compared to 2D culture. Furthermore, the expression of exosomes biogenesis markers, glycolysis genes, and miRNAs (miR-10, 19a, 19b, 21,132, and 377) were upregulated in the exosomes from 3D bioreactor culture compared to static 2D culture [93].

Additionally, the secretory function of MSCs undergoes alteration under the influence of biochemical reagents, consequently imparting specific biological functions to exosomes. Li et al. [94] treated MSCs with lipopolysaccharide (LPS) to generate exosomes with immunomodulatory and anti-inflammatory functions. When exosomes derived from LPS-pre-MSC (LPS pre-Exos) were applied in the sciatic nerve injury model and inflammatory model, LPS pre-Exos promoted functional recovery, axonal regeneration, remyelination, and M2 macrophage polarization. The potential mechanism involved high enrichment of TNF stimulated gene-6 (TSG-6) in LPS pre-Exos, which exerted inhibitory effects on NF-κB and NOD-like receptor protein 3 (NLRP3) during macrophage polarization (Figure 4B). The immunosuppressive drug tacrolimus (FK506), in conjunction with ADMSCs), was employed in cell therapy for organ transplantation and vascularized composite allotransplantation [95,96]. Kuo et al. [97] locally administered exosomes derived from FK506-treated ADMSCs (ADMSC-F-exo) in the sciatic nerve crush injury model, revealing a significant reduction in the autophagy of macrophages in the spinal cord segment (Figure 4C). The phenomenon may be associated with the involvement of heat shock protein family A member 8 (HSPA8) and eukaryotic translation elongation factor 1 alpha 1 (EEF1A1) in exosome-mediated autophagy.

### 7.2. Direct Engineering Approaches of Exosomes

**Figure 5:**
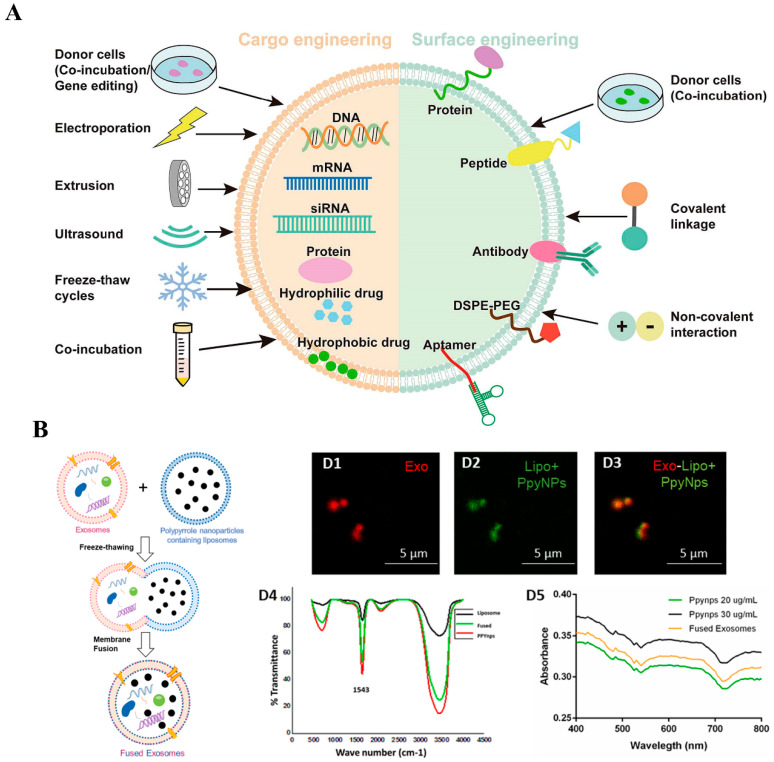
Engineering approach for exosome application in peripheral nerve injury. (**A**) Engineering exosomes to achieve cargo engineering and surface engineering through co-incubation, extrusion, electroporation, sonication, freeze and thaw cycles, liposome-based transfection, and click chemistry. Adapted with permission from [98], copyright 2023, Springer Nature. (**B**) Membrane fusion of BMSC-derived exosomes and PpyNps containing liposomes by the freeze−thawing mechanism. Adapted with permission from [99], copyright 2021, Elsevier.

Although exosomes can be engineered at the cellular level through culture condition and biochemical reagents, hydrophobic and hydrophilic drugs, fluorescent probes, and siRNA face challenges in being loaded into exosomes due to changes in cellular uptake. To overcome the limitations of cell engineering strategies on the drug delivery, fluorescence imaging, and gene therapy potential of natural exosomes, some studies have explored direct engineering approaches for exosomes. These engineered approaches include incubation with cargos and membrane permeabilizer, extrusion, electroporation, sonication, freeze and thaw cycles, liposome-based transfection, and click chemistry [98] (Figure 5A).

In brain injury and spinal cord injury, the application of directly engineered exosomes in nerve regeneration has been extensively documented. However, limited research has been conducted on the direct engineering of exosomes in peripheral nerve regeneration. In the diabetic peripheral neuropathy, Singh et al. [99] devised a combinatorial approach involving the fusion of exosomes derived from BMSCs with liposome-encapsulated polypyrrole nanoparticles (PpyNPs) to provide biochemical and electrical signals. The combination was achieved by subjecting exosomes derived from BMSCs and liposomes loaded with PpyNPs to ten cycles of freeze–thaw (Figure 5B). The composite system offered biochemical and electrical signal stimulation for nerve regeneration, as exosomes contained biochemical components, while PpyNPs can induce electrical conduction for nerve regeneration. Intramuscular injection of the delivery system contributed to the normalization of nerve conduction velocity and compound muscle action potentials, along with the restoration of gastrocnemius muscle morphology, muscle mass, and integrity. Remarkably, engineered exosomes exhibited paracrine effects in controlling hyperglycemia and weight loss.

## 8. MSC Exosomes Combined with Biomaterials Promote Peripheral Nerve Regeneration

Recent studies indicated that the local application of nerve conduits and hydrogels plays a facilitative role in promoting peripheral nerve regeneration. Applying exosomes derived from mesenchymal stem cells directly to the site of peripheral nerve injury bypasses metabolic clearance challenges linked to systemic delivery. The integration of exosomes into nerve conduits and hydrogels facilitates localized and sustained release, mitigating clearance by the host organism.

### 8.1. Nerve Conduit Incorporating Exosomes

**Figure 6:**
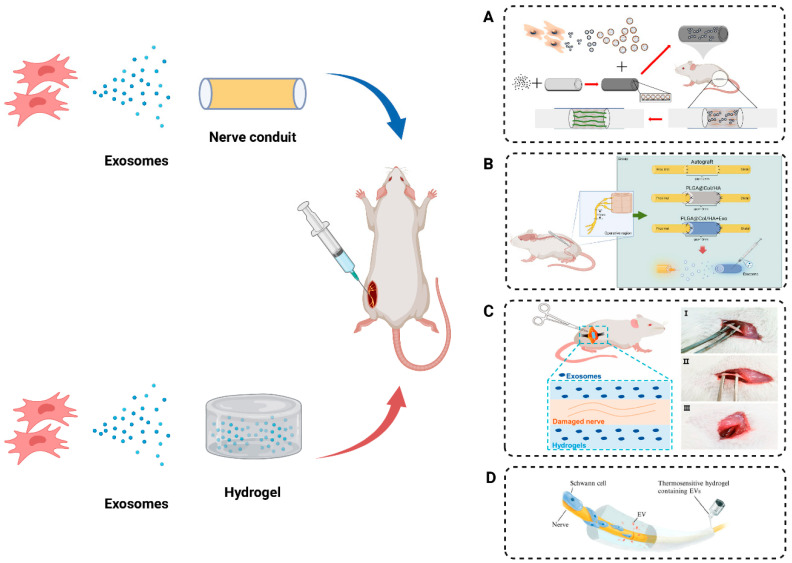
Approaches for MSC exosomes’ delivery to the peripheral nerve injury. (**A**) The polydopamine-modified chitosan nerve conduit was loaded with BMSC-derived exosomes to bridge 2 mm defect of sciatic nerve in rat. Adapted with permission from [106], copyright 2022, Wolters Kluwer Medknow Publications. (**B**) hUCMSC-derived exosomes combined with 3D composite conduit (PLGA@Col/HA) repaired long-gap peripheral nerve injury, promoting motor function recovery and reducing muscle atrophy. Adapted with permission from [107], copyright 2023, Wolters Kluwer Medknow Publications. (**C**) Different concentrations of HAMA solution mixed with hUCMSC-derived exosomes were injected into the injured nerve, and after curing with ultraviolet light, the hydrogels with different mechanical properties were formed at the sciatic nerve crush injury site (I, uninjured rat sciatic nerve; II, injured sciatic nerve; and III, injured nerve embedded in hydrogel). Adapted with permission from [109], copyright 2022, Frontiers. (**D**) Exosomes released from the PALDE hydrogel promoted peripheral nerve regeneration by directly stimulating neurite outgrowth and indirectly promoting Schwann cell migration and proliferation. Adapted with permission from [111], copyright 2022, AIP Publishing.

The nerve conduit utilizes a tubular support structure to complement autologous nerve transplantation as a therapeutic technique and has extensive applications in peripheral nerve regeneration. Nerve conduits can prevent the misdirection of nerve fibers and inhibit inward tissue growth, thereby reducing the occurrence of neuromas. However, the reparative capacity of a single nerve conduit transplantation for peripheral nerve injury is limited. Local injection of MSC-derived exosomes at the residual end of nerve injuries has been proven effective in promoting nerve repair and yielding favorable outcomes [100,101,102,103]. In this context, our research group previously developed a biodegradable chitosan nerve conduit. Subsequently, Rao et al. [104]. demonstrated that local injection of dental pulp mesenchymal stem cell (DPMSC)-derived exosomes into the nerve conduit significantly increased the quantity and diameter of sciatic nerve fibers. The promotion of myelin sheath formation consequently facilitated the restoration of muscle function, nerve conduction, and motor function. Similarly, Zhang et al. [105] locally injected exosomes derived from BMSCs building on the earlier development of rGO-GelMA-PCL nerve conduits for repairing peripheral nerve injury. The results indicated superior outcomes, including increased numbers of newly formed blood vessels and axons at the injury site, as well as the recovery of nerve function compared to using nerve conduit alone. Nevertheless, the injected exosomes may leak from the conduit interstice due to limb movement. To better preserve and sustain the release of exosomes, Li et al. [106] modified chitosan conduits with dopamine and subsequently loaded exosomes to repair a 2 mm sciatic nerve defect in rats, resulting in accelerated nerve healing and improved nerve function (Figure 6A). Furthermore, researchers successfully repaired a 10 mm peripheral nerve injury using a 3D composite nerve conduit composed of synthetically derived polymers (electrospun hollow poly (lactic-co-glycolic acid) tube with collagen/hyaluronic acid inner sponge) in conjunction with exosomes from UCMSCs [107] (Figure 6B). The studies on the combined application of nerve conduit and MSC-derived exosomes in peripheral nerve injury were listed in Table 3.

### 8.2. Hydrogels Incorporating Exosomes

Hydrogels, characterized by highly hydrophilic three-dimensional network structures, have attracted considerable attention owing to their tailorable release properties, transparency, biocompatibility, and exceptional mechanical flexibility [108]. Research indicated that the hardness, conductivity, and composition of hydrogels are pivotal factors influencing the delivery of drugs or molecules and tissue repair. For instance, the hardness of photoresponsive hyaluronic acid methacrylate hydrogels plays a vital role in nerve injury repair [109] (Figure 6C). Compared to rigid hydrogels loaded with exosomes, soft hydrogels enable the rapid release of exosomes. These inhibited the infiltration of macrophages into damaged nerves and promoted the expression of pro-inflammatory factors IL-1β and TNF-α, thereby enhancing peripheral nerve repair. Conductive hydrogels, possessing mechanical and bioelectrical characteristics similar to neural tissue, are found to have extensive applications in neural regeneration and motor function recovery following peripheral nerve injuries. Yang et al. [110] applied electroconductive hydrogels (ECH) loaded with exosomes derived from BMSCs to treat diabetic peripheral nerve damage. ECH-Exos promoted Schwann cell adhesion and migration, while the exosomes in the delivery system regulated M2 macrophage polarization through the NF-κB pathway, alleviating ammation and pain associated with diabetic peripheral nerve damage. Additionally, ECH-Exos enhanced myelinated axon regeneration through the MEK/ERK pathway in vitro and in vivo, ameliorating muscle denervation and contributing to enhanced functional recovery. Temperature-sensitive hydrogels undergo phase changes or swelling/collapse in response to temperature variations, offering improved local drug permeation, optimal spatial and temporal control for drug delivery. After microsurgery, nerve regeneration is slow. To expedite peripheral nerve regeneration, researchers applied ADMSC exosomes in thermosensitive hydrogels. These hydrogels rapidly solidify, maintaining a high concentration of exosomes around the nerves [111] (Figure 6D). Similarly, thermosensitive hydrogel composed of hydroxyethyl chitosan/β-glycerophosphate exhibited excellent injectability, structural stability, and temperature sensitivity. In vivo experiments demonstrated that hydrogels loaded with exosomes from ADMSCs enhanced exosome retention and slow release [112].

## 9. Conclusions and Future Perspectives

Mesenchymal stem cell (MSC)-derived exosomes have shown significant potential as a nanomedicine in supporting peripheral nerve regeneration. The remarkable therapeutic effects attributed to these nanovesicles are due to the abundant bioactive components encapsulated within them, such as growth factors, anti-inflammatory molecules, and miRNAs. The synergistic actions of these constituents contribute to vascular regeneration at the site of nerve injury, axonal regeneration, regulation of inflammatory responses, promotion of neuronal survival, and stimulation and guidance of the regeneration and repair processes in the surrounding nerves. Physiological stimuli or small molecule modulators have been demonstrated to enhance the secretion of MSC exosomes. However, a comprehensive investigation into the physicochemical properties, composition, and functions of MSC-derived exosomes obtained through these approaches is imperative. Due to the limitations associated with the sole application of MSC exosomes, several studies have combined MSC exosomes with biomaterials or utilized them as carriers for genes or drug delivery. Therefore, it is crucial to further elucidate the collaborative actions and targeted delivery mechanisms of MSC exosomes.

With an increasingly profound understanding of the therapeutic mechanisms associated with MSC-derived exosomes, researchers are continuously striving to optimize the exosome preparation process to enhance their therapeutic efficacy, stability, and controllability. This endeavor involves exploring more efficient extraction methods, refining techniques for exosome loading, and designing personalized treatment regimens. Concurrently, with the advancement of clinical investigations, there is a growing opportunity to validate the efficacy of exosomes in diverse types of nerve injuries, thereby providing robust support for their broader application in clinical practice (NCT03384433, NCT06138210, NCT05035134, NCT05370105, Source: ClinicalTrials.gov). For example, Civelek et al. [113] conducted a preliminary clinical study of MSC-derived exosomes in the treatment of a patient with total radial nerve. Consequently, MSC-derived exosomes, being an innovative and promising therapeutic modality, bring new treatment prospects and a more sustainable trajectory for the recovery of patients with peripheral nerve injuries in the future.

## Figures and Tables

**Table 1:** Comparison of exosome isolation techniques.

Isolation Technique	Isolation Principle	Potential Advantage	Potential Disadvantage
Ultracentrifugation	Density, Size	Gold standard method Low cost and high purity Large sample preparation Minimal expertise requirement	Expensive equipment requirement Labor intensive Low yield and biological activity Protein aggregation Time consuming
Ultrafiltration	Molecular weight, Size difference	Fast operation Good portability Low equipment cost	Exosomes loss due to clogging and membrane trapping Moderate purity
Immunoaffinity capture	Affinity	Easy operation High specificity Hight purity	Expensive antibodies Extra steps for exosomes elution after binding to antibodies Low sample capacity and yield
Polymer precipitation	Solubility or dispersibility, Surface charge	Easy operation High yield Large sample preparation Ordinary equipment requirement	Affecting downstream analysis Low purity Long processing time Require complicated clean-up
Size exclusion chromatography	Molecular weight, Size difference	Fast operation High purity High yield Keep native function and structure	Contamination of similarly sized proteins Require equipment for exosomes enrichment Special device requirement
Ion exchange chromatography	Charge	Easy to use High purity	Specific isolation unavailable
Microfluidics-based techniques	Acoustic, density, electrophoretic, electromagnetic immunoaffinity, size	Easy automation and integration Fast operation High portability High purity	Low sample capacity Lack of method validation and standardization
Membrane-based isolation	Surface properties	Fast operation High affinity Successful with greater exosomes	Contamination of similar surface property molecules Low purity

**Table 3:** Nerve conduit combined with MSC exosomes repair PNI.

Nerve Conduit	Source Cell	Exosome Isolation Methods	Exosome Concentration	Effects	References
Chitin conduit	hGMSC	Ultracentrifugation	100 μg/mL (in vitro), 10 μg exosomes in 10 μL PBS (in vivo)	Increased the number and diameter of nerve fibers and promoted myelin formation	[104]
Polydopamine-modified chitin conduit	BMSCs	Ultracentrifugation	N/A	Promoted the functional recovery of injured peripheral nerves	[106]
rGO-GelMA-PCL nerve conduit	BMSCs	Ultracentrifugation	20 μg exosomes in 20 μL PBS (in vitro and in vivo)	Increased the number of newly formed vessels and axonal sprouts	[105]
Silicone tube	ADMSCs	Ultracentrifugation	20 μg/mL (in vitro), 1 mg/mL in alginate solution (in vivo)	Improved the function recovery of gastrocenemius muscles and promoted nerve regeneration	[100]
Silicone tube	ADMSCs	Ultracentrifugation	5, 10, 20 μg/mL (in vitro), 20 μg/mL (in vivo)	Improved axon regeneration, myelination, and restoration of denervation muscle atrophy.	[101]
Electrospun hollow poly (lactic-co-glycolic acid) tube with collagen/hyaluronic acid	hUCMSCs	Ultracentrifugation	PBS containing 10 μL of exosomes (10 mg/mL in PBS)	Promoted peripheral nerve regeneration and restoration of motor function.	[107]
Core-shell silk-fibroin/poly-l-Lactic acid nerve conduit	Human endometrial stem cells	Ultracentrifugation	N/A	Enhanced the regeneration process of axons and improved the functional recovery of rat sciatic nerve defects	[102]
Biosynthetic cellulose conduit	ADMSCs	Ultracentrifugation	4 μL exosome solution (1.9 × 10^8^ exosomes/μL)	Produced comprehensive and durable repair of peripheral nerve defects	[103]
PHBV nanofibrous scaffold	ADMSC	Ultracentrifugation	5, 10, 20 μg/mL (in vitro), 2 mg for each rat (in vivo)	Facilitated the regeneration of injured sciatic nerves	[91]
